# Impact of *Leishmania mexicana* Infection on Dendritic Cell Signaling and Functions

**DOI:** 10.1371/journal.pntd.0003202

**Published:** 2014-09-25

**Authors:** Irazú Contreras, José A. Estrada, Hannah Guak, Caroline Martel, Alborz Borjian, Benjamin Ralph, Marina T. Shio, Sylvie Fournier, Connie M. Krawczyk, Martin Olivier

**Affiliations:** 1 Department of Microbiology and Immunology, McGill University, Montréal, Quebec, Canada; 2 Neurochemistry Laboratory, Faculty of Medicine. Universidad Autónoma del Estado de México, Toluca, Estado de México, México; 3 McGill TB International Centre and the Research Institute of the McGill University Health Centre, Montréal, Quebec, Canada; Louisiana State University, United States of America

## Abstract

*Leishmania* parasites have the ability to modify macrophage signaling pathways in order to survive and multiply within its mammalian host. They are also known to invade other cells including neutrophils, fibroblasts and dendritic cells (DCs). DCs have an important role in immunity as the link between innate and adaptive immunity, necessary for the development of an effective response; however, the impact of *Leishmania mexicana* infection on DCs has been poorly studied. Herein, we report that *Leishmania* infection rapidly induced DC protein tyrosine phosphatases activity, leading to MAP kinases inactivation. In line with this, *L. mexicana* was found to decrease the nuclear translocation of transcription factors such as AP-1 and NF-κB. Concomitantly, *L. mexicana*-infected DCs showed reduced expression of several surface antigen-presenting and co-stimulatory molecules upon LPS stimulation. *Leishmania*-induced interference on DC maturation was further reflected by their reduced capacity to present OVA antigen to OVA-specific T cells, as shown by abrogation of IL-2 production by the T cells. Collectively, our data revealed that DC infection by *L. mexicana* appears to affect the cellular and immunological mechanisms necessary for the development of an effective and protective immune response, therefore favouring the survival and propagation of the parasite within its host.

## Author Summary

Parasites of the *Leishmania* genus have developed many strategies to survive inside their host. Initially, they were only considered capable of infecting macrophages; however, it has been observed that *Leishmania* is able to infect other cell types, such as fibroblast, neutrophils and dendritic cells (DCs). DCs are well known for their antigen-presentation capabilities, and they are considered as the fundamental bridge between the innate and adaptive immune responses. In this study, we attempted to elucidate the effect of *L. mexicana* promastigotes on DCs. Our results showed that *L. mexicana* inactivates signaling cascades responsible for the expression of immune effector molecules, such as cytokines, concomitantly with the activation of protein phosphatases in the host. Furthermore, we observed that promastigote-infected cells had lower expression of MHC and co-stimulatory molecules on their surface, as well as decreased antigen-presentation capacity. In conclusion, our study showed that *Leishmania* parasites are able to inactivate the immunological mechanisms of DCs, as they do in macrophages, in order to survive inside its host.

## Introduction

Leishmaniasis refers to a group of diseases caused by protozoan parasites of the *Leishmania* genus, transmitted by phlebotomine female sandflies [1], [2]. This infection is characterized by three main clinical manifestations: the self-healing cutaneous Leishmaniasis (CL); disfigurating, localized muco-cutaneous Leishmaniasis (MCL), and the life-threatening visceral Leishmaniasis (VL). These diseases are endemic in areas of the tropics, subtropics and southern Europe [1]. *Leishmani*a parasites reside, multiply, and change from the promastigote stage to the amastigote stage inside their primary host cell: the macrophage. Importantly, this group of pathogens has evolved multiple mechanisms to subvert macrophage functions by altering their signaling pathways in order to survive and propagate within its mammalian host [3]. Although macrophages are considered as the canonical host cells, neutrophils [4], fibroblasts [5], and dendritic cells (DCs) [6], [7] have also been reported to be susceptible to *Leishmania* infection. However, little is known regarding the impact that *Leishmania* has on DC signaling pathways and their immunological functions.

DCs are professional antigen presenting cells (APC), which in peripheral non-lymphoid tissues sit in an immature state capable of antigen uptake and processing [8]. They are also critical for the induction of immunological tolerance, as well as for the regulation of T cell-mediated immune responses [9]. The maturation process of DCs consists of: i) increased expression of MHC and co-stimulatory molecules, such as CD40, B7.1, B7.2 and CD54; ii) down-regulation of antigen capture and phagocytic capacity; iii) enhanced cytokine secretion and iv) expression of different chemokine receptors [10]. Uptake of antigens by DCs is mediated by different groups of receptors, such as Fc-receptors [6], C-type lectin receptors (CLR), which recognize glycoproteins, and Toll-like receptors [11]. All of these receptors are able to recognize a wide variety of microorganisms, including *Leishmania* parasites [12], [13].

Despite the well-known role of DCs as a link between the innate and adaptive immune responses, the impact of *Leishmania* infection on DC functional activities and signaling pathways remains very controversial and largely unexplored. A better understanding on how *Leishmania* may influence the functions of these cells could permit the development of better approaches to strengthen DC responses for the control of *Leishmania* infection. In this study, we investigated the impact of *L. mexicana* promastigote infection on DC maturation and antigen presentation capacities using the DC2.4 cell line [14]. Our study revealed impairment of MAPK signaling in *L. mexicana*-infected DCs. Dephosphorylation of molecules associated with MAPK signaling was accompanied by augmented protein tyrosine phosphatase (PTP) activity and concomitant alteration of the transcription factors AP-1 and NF-κB, events that have been also observed in *Leishmania*-infected macrophages [12], [15]–[18]. Finally, we also observed that *L. mexicana*-infected, OVA peptide-pulsed DCs, were less efficient in their capacity to present antigen to OVA-specific T cells, as reflected by an almost complete absence of IL-2 production by the latter. In part, this antigen presentation defect could be due to a decreased expression of certain antigen presentation molecules and co-stimulatory molecules observed in infected cells. Collectively, the present study clearly establishes that *Leishmania*-infected DCs are strongly compromised in their capacity to develop an effective adaptive immune response, thereby favouring a successful infection and further propagation of the parasite within its host.

## Materials and Methods

### Cell culture, infection and stimulation

Immortalized murine (C57BL/6) bone marrow-derived dendritic cells of the DC2.4 cell line [14] were maintained at 37°C in 5% CO_2_ in RPMI medium supplemented with 10% heat inactivated FBS (Invitrogen, Burlington, ON, Canada), 100 U/ml penicillin-100 µg/ml streptomycin and 2 mM of L-glutamine (Wisent, St-Bruno, QC, Canada), 10% HEPES (Sigma-Aldrich, St-Louis MO, USA), 10% essential amino acids (Invitrogen) and 50 µM of 2β-mercaptoethanol (Sigma- Aldrich). Bone marrow derived DCs (BMDCs) were differentiated in the presence of GM-CSF (20 ng/ml) in complete DC medium (RPMI 1640 containing 10% FCS, 100 U/ml penicillin/streptomycin, 0.05 mM 2β-mercaptoethanol and 2 mM L-glutamine). On days 8 and 10 of culture, DCs were washed in complete DC medium containing 5 ng/ml GM-CSF and infected with *Leishmania mexicana* stationary phase promastigotes. MHC-II-OVA-specific T cell hybridome cells (MF2.9) were grown and maintained in the same medium used for DCs by tri-weekly passage. *Leishmania mexicana* promastigotes were grown and maintained at 25°C in SDM-79 culture medium supplemented with 10% FBS by bi-weekly passage. *Leishmania mexicana* promastigotes were grown in SDM medium (10% FBS) [19] for 7 days to reach stationary phase. DC2.4 cells were infected with stationary phase promastigotes at a parasite to DC ratio of 20∶1 (infection was also performed at 5∶1 and 10∶1 ratios (data not shown); however, a high infection ratio (20∶1) guaranteed at least 80% of infected cells (Figure S1). BMDCs were infected with promastigotes (20∶1 ratio), for the time specified in each figure legend. The ratio of *Leishmania*:BMDC specified, yielded approximately 85% of cellular infection, as illustrated in Figure S1. To assess the parasite load, BMDCs were plated on coverslips and infected with the indicated ratio of promastigotes. After 18 hr of infection, coverslips were stained with Diff-Quik (Siemens) and images were taken using the Zeiss Axio lab.A1 microscope with an Infinity 1 camera system at the Goodman Cancer Research Centre Histology Core, McGill University. DC stimulation was performed with 100 ng/ml of LPS (Sigma-Aldrich) used at the times specified in each figure legend and remained throughout the infection time.

### Western blot

Infected and uninfected DCs (1×10^6^) were washed 3 times with Phosphate Buffered Saline (PBS) and lysed with cold lysis buffer (50 mM Tris (pH 7), 1 mM, 0.1 mM EGTA, 0.1% 2-mercaptoethanol, 1% IGEPAL, 40 µg/ml aprotinin and 20 µg/ml of leupeptin). Proteins were dosed by Bradford's method (Bio-Rad, Hercules CA, USA), and 60 µg of proteins were separated by SDS-PAGE, and transferred onto PVDF membranes (GE healthcare, Piskataway NJ, USA). Membranes were blocked in 5% non-fat dry milk, washed and incubated overnight (ON) with anti-p-ERK, p-JNK, and p38 antibodies (Cell signaling, Ipswich, MA, USA). After washing, membranes were incubated 1 hr with α-rabbit HRP- conjugated antibody (Sigma-Aldrich), and developed by chemiluminescence.

### In gel PTP assay

In gel PTP assay was performed as previously described [20]. Briefly, Poly (Glu-Tyr) substrate was tyrosine phosphorylated by ON incubation with 10 µg of GST-FER protein kinase and 150 µCi of γ-^32^ P-dATP. The radiolabeled-phosphorylated substrate was incorporated in a 10% SDS-PAGE at a concentration of 2×10^5^ CPM/ml. DC extracts were prepared as described for WB and denatured by SDS. After electrophoresis, gels were incubated ON in buffer A (50 nM Tris-HCL pH 8.0, 20% isopropanol), then gels were washed twice with buffer B (50 nM Tris-HCL pH 8.0, 0.3% 2β-mercaptoethanol), followed by full protein renaturation in buffer B containing 6 M guanidine and 1 mM EDTA. The gels were washed twice in buffer C (50 mn Tris-HCL pH 8.0, 0.3% 2β-mercaptoethanol, 1 mM EDTA and 0.04% tween 20). Final renaturation was obtained by 18 hr incubation with buffer C. Gels were dried and exposed to X-ray film.

### pNPP assay

Infected and uninfected DCs were lysed in PTP buffer (50 mM Tris-HCL pH 7.0, 0.1 mM EDTA, O.1 mM EGTA, 0.1% 2β-mercaptoethanol, 1% IGEPAL, 25 µg/ml aprotinin, and 25 µg/ml leupeptin) and kept on ice for 45 minutes, vortexing every 15 minutes. Lysates were cleared by centrifugation, and protein content was measured by Bradford's method. 10 µg of protein extract were incubated in phosphatase reaction buffer (50 mM HEPES pH 7.5, 0.1% β-mercaptoethanol, 10 mM para- nitrophenylphosphate (pNPP)) for 30 minutes. OD was read at 405 nm.

### Electrophoretic Mobility Shift Assay (EMSA)

2×10^6^ DC 2.4 cells were infected as mentioned above, washed with PBS to remove non-internalized parasites, and processed for nuclear extraction as previously described [16], [21]. Briefly, DCs were collected in 1 ml of cold PBS, centrifuged and pellets were resuspended in 400 µl of ice-cold buffer A (10 mM HEPES, 10 mM KCl, 0.1 mM EDTA, 0.1 mM EGTA, 1 mM DTT and 0.5 mM of PMSF) and incubated 15 min on ice. Twenty five µl of IGEPAL 10% (Sigma-Aldrich) were added, and the samples were vortexed for 30 sec. Nuclear proteins were pelleted by centrifugation and resuspended in 50 µl of cold buffer C (20 mM HEPES, 400 mM NaCl, 1 mM EDTA, 1 mM EGTA, 1 mM DTT and 0.5 mM PMSF). Protein concentrations were determined by a Bradford's method (Bio-Rad). 6 µg of nuclear proteins were incubated for 20 min at room temperature with 1 µl of binding buffer (100 nM Hepes pH 7.9, 8%, v/v glycerol, 1% w/v Ficoll, 25 mM KCl, 1 mM DTT, 0.5 mM EDTA, 25 mM NaCl, and 1 µg/µl BSA) and 200 ng/µl of poly (dI-dC), 0.02% bromophenol blue and 1 µl of γ- P^32^ labeled oligonucleotide containing a consensus sequence for AP-1 binding complexes (5′-CGTTTGATGACTCAGCCGGAA-3′) (Santa Cruz Biotechnology Inc, Santa Cruz CA, USA), and NF-κB (5′-AGTTGAGGGGACTTTCCCAGGC-3′) (Santa Cruz Biotechnology Inc). After incubation, DNA-protein complexes were resolved by electrophoresis in a non-denaturing 5% (w/v) polyacrilamide gel. Subsequently, gels were dried and autoradiographed. Competition assays were conducted by adding a 100-fold molar excess of homologous unlabeled AP-1 or NF-κB oligonucleotides, or the non-specific competitor sequence for SP-1 binding (5′-ATTCGAATCGGGGCGGGGCGAGC-3′).

### Flow cytometry (cell surface staining)

0.5×10^6^ DCs were plated in 10 cm dishes, recovered after 24 hr of infection (*L. mexicana*) or 24 hr of stimulation (LPS 100 ng/ml) and blocked with 2.4G2 (anti-FcγR) in staining buffer (1% bovine serum albumin (BSA) and 0.1% NaN_3_ in PBS 1×). Samples were then labeled with primary biotinylated antibodies against cell surface molecules (anti-mouse MHC I and MHC II (BD biosciences, Mississauga ON, Canada)), and subsequently, labeled with secondary antibody, PeCy5-conjugated streptavidin (eBiosciences, San Diego CA, USA) and fluorochrome-conjugated antibodies against cell surface molecules (anti-mouse B7.1, B7.2, CD11b and CD40 (eBiosciences). Finally, samples were acquired on a FACS calibur (BD biosciences) and analyzed with Cell Quest software (BD biosciences). Isotype controls were used to set quadrants. BMDCs were harvested and blocked with 2.4G2 in staining buffer; cells were stained with fluorochrome-conjugated antibodies for surface markers (CD11b, CD11c, CD40, CD80, CD86, and MHC-II, from BD biosciences and eBiosciences). For MHC-I staining, a primary biotinylated antibody was used (BD biosciences), which was subsequently labeled with APC-conjugated streptavidin (Cedarlane, Burlington, ON, Canada). After staining, samples were fixed with 2% formaldehyde and acquired on a FACSCanto II flow cytometer. Analysis was performed using the FlowJo software. Analysis gates were based on CD11c^+^ cells.

### IL-12 mRNA expression analysis

RNA was extracted from DCs using TRIzol reagent (Invitrogen). Reverse transcription was performed using oligodt (Invitrogen). Quantitative real-time PCR (RT-PCR) was performed in a Corbbet research rotorgene (Corbett life science, Sydney, Australia), using Bio-Rad SYBR green qPCR super mix (Bio-Rad) and 0.4 µM IL-12 p40/GAPDH primers (GAPDH: 5′-CGG ATT TGG CCG TAT TGG GCG CCT-3′ and 3′-ACA TAC TCA GCA CCG GCC TCA CCC-5′; IL-12: 5′- GGA AGC ACG GCA GCA GAA TA-3′ and 3′-AAC TTG GAG AAG TAG GAA TGG-5′) in a final volume of 25 µl. The qPCR program included 2 min at 50°C; 3 min at 95°C; 45 cycles of 20 sec at 95°C, 30 sec at 60°C and 20 sec at 72°C followed by a melting curve. The annealing temperature for all primers was 60°C.

### Co-culture assays (DCs-T cells)

2×10^4^ DC2.4 cells were plated in 96 well plates and either infected with *L. mexicana* promastigotes or LPS-stimulated at the times specified in each figure legend. After infection or stimulation, cells were washed 3 times with PBS to remove all non-internalized parasites and loaded with 2 mg/ml of OVA (Sigma-Aldrich) for 2 hr. After that, 1×10^5^ MF2.9 MHC-II- specific-OVA-hybridome T cells were co-cultured with DCs ON. The following day, plates were centrifuged and supernatants were collected and frozen at −20°C until they were used to measure IL-2 production by T cells.

For primary co-cultures, BMDCs were plated at 2×10^6^ cells per well in a 24-well, non-tissue culture treated plate and infected with *L. mexicana* at a 20∶1 ratio. After 6 hours, parasites were gently washed with PBS. Each well received 2 mg of OVA and complete DC media, with or without 100 ng/ml of LPS, for an additional 18 hrs. DCs were collected and co-cultured in 96-well tissue culture treated plates with CD4^+^ T cells, at a 1∶10 ratio of DCs to T cells (2×10^4^∶2×10^5^). CD4^+^ T cells were isolated from C57BL/6 OTII mice using the EasySep Mouse CD4^+^ T cell Isolation Kit (Stemcell). To measure intracellular cytokine production, the cells were restimulated with 50 ng/mL of PMA and 500 ng/mL of ionomycin, for 4 hours, with Brefeldin A present for the last two hours. The cells were then stained for the surface molecules CD44 and CD4, and intracellular IFN-γ. The samples were acquired using a FACSCanto II (BD Bioscience) and the data was analyzed with the FlowJo software.

### ELISA

NUNC maxisorb 96 well plates (Nalge NUNC, Richester NY, USA) were coated with 100 µl/well of capture antibody (SET TO GO kits from eBiociences) ON and blocked with 200 µl/well with assay diluent solution 1 hr at RT. After blocking, 100 µl of standard proteins or samples were added to each well and incubated for 2 hr. After 5 washes, 100 µl/well of detection antibody were added and incubated 1 hr at RT. For IL-2, IL-10, IL-12p40, and IL-12p70 detection, 100 µl/well of Avidin-HRP were added and incubated for 30 min. Finally, 100 µl/well of substrate solution were added for 15 min to develop color. 50 µl of stopping solution were added and plates were read at 450 nm in an ELISA reader. IL-2, IL-10, IL-12p40, and IL-12p70 concentrations were measured and analyzed using a standard curve provided by the IL-2, IL-10, IL-12p40, and IL-12p70 commercial kits.

### Statistical analyses

Statistically significant differences were identified using the ANOVA test and an unpaired T test with Welch correction on the Prism program (version 5). Values of P≤0.05 were considered statistically significant. All data are presented as mean ± SEM.

## Results

### *L. mexicana* promastigotes activate DC protein tyrosine phosphatases

**Figure 1:**
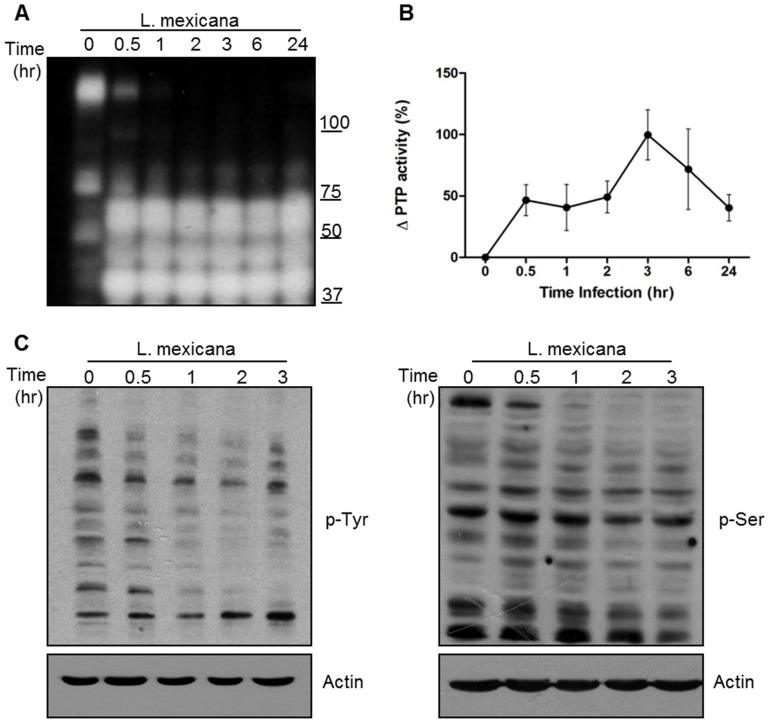
*L. mexicana* promastigotes activate PTPs in DCs. (**A**) In gel assay of cell extracts from DC 2.4 cells infected with *L. mexicana* promastigotes for 0.5, 1, 2, 3, 6 and 24 hr. (**B**) pNPP assay of cell lysates of DC 2.4 cells infected with *L. mexicana* promastigotes for 0.5, 1, 2 3, 6 and 24 hr. (**C**) Western blot analysis of cell lysates from *L. mexicana*-infected DC2.4 cells (0.5, 1, 2 and 3 hr) and blotted against p-ser (right panel) or p-tyr (left panel). Actin was used as loading control. In all cases, DCs were infected in a 1∶20 cell-parasite ratio. One representative experiment of three is shown.

Activation of PTP is an important negative regulatory mechanisms employed by *Leishmania* to subvert macrophages signaling favoring its survival within the host cell. In this regard, we have previously reported that SHP-1, a critical PTP of macrophages, is activated upon *Leishmania* infection [12], [18], [22]. Thus, we explored this phenomenon in *L. mexicana*-infected DCs from the DC2.4 cell line. To this end, we performed an in gel phosphatase assay, where addition of a radiolabeled peptide into a SDS-PAGE gel allows detection of the PTP profile as well as their activity. This technique allows only the detection of mammalian PTPs, and does not detect the parasites' PTPs, as shown in Figure S2. As soon as 30 min post-infection, there was appearance of bands corresponding to active PTPs that were not present in the uninfected control (Figure 1A). This PTP activity was maintained after 24 hr of infection. Furthermore, we performed a PTP activity assay using the substrate pNPP, which is a more sensitive method to determine PTP activity. The activity of PTPs increased ∼50% as soon as 30 min post- infection and reached its maximum peak at 3 hr (Figure 1B). The increase, as well as the decrease, of PTP activity observed in the pNPP assay might be the result of transitory regulation of the PTPs. These modifications are not observed in the in-gel assay, due to the lower sensitivity of this method. In addition, as shown is Figure S2, LPS stimulation has no effect on the in-gel PTP activity profile.

As phosphatases regulate kinase activity, we performed WB analysis to evaluate the profile of protein phosphorylation on serine and tyrosine residues. As shown in Figure 1C, upon *Leishmania* infection there was decreased phosphorylation on serine (right panel) and tyrosine (left panel) residues, suggesting that this lack of phosphorylation was indeed due to the increased phosphatase activity observed in both assays.

### *Leishmania* infection inhibits DC signaling pathways

Activation of cell signaling pathways is crucial for the production of the cytokine/chemokine networks that control the course of infectious diseases, such as Leishmaniasis. *Leishmania* parasites have the ability to suppress important intracellular signaling events in macrophages [3]. However, limited information is available regarding the effects on DC signaling pathways upon *Leishmania* infection. Nonetheless, a recent report has showed that infection of DCs with *L. amazonensis* amastigotes resulted in alteration of the STAT-2 pathway, with a concomitant reduction in the production of IL-12 [23].

**Figure 2:**
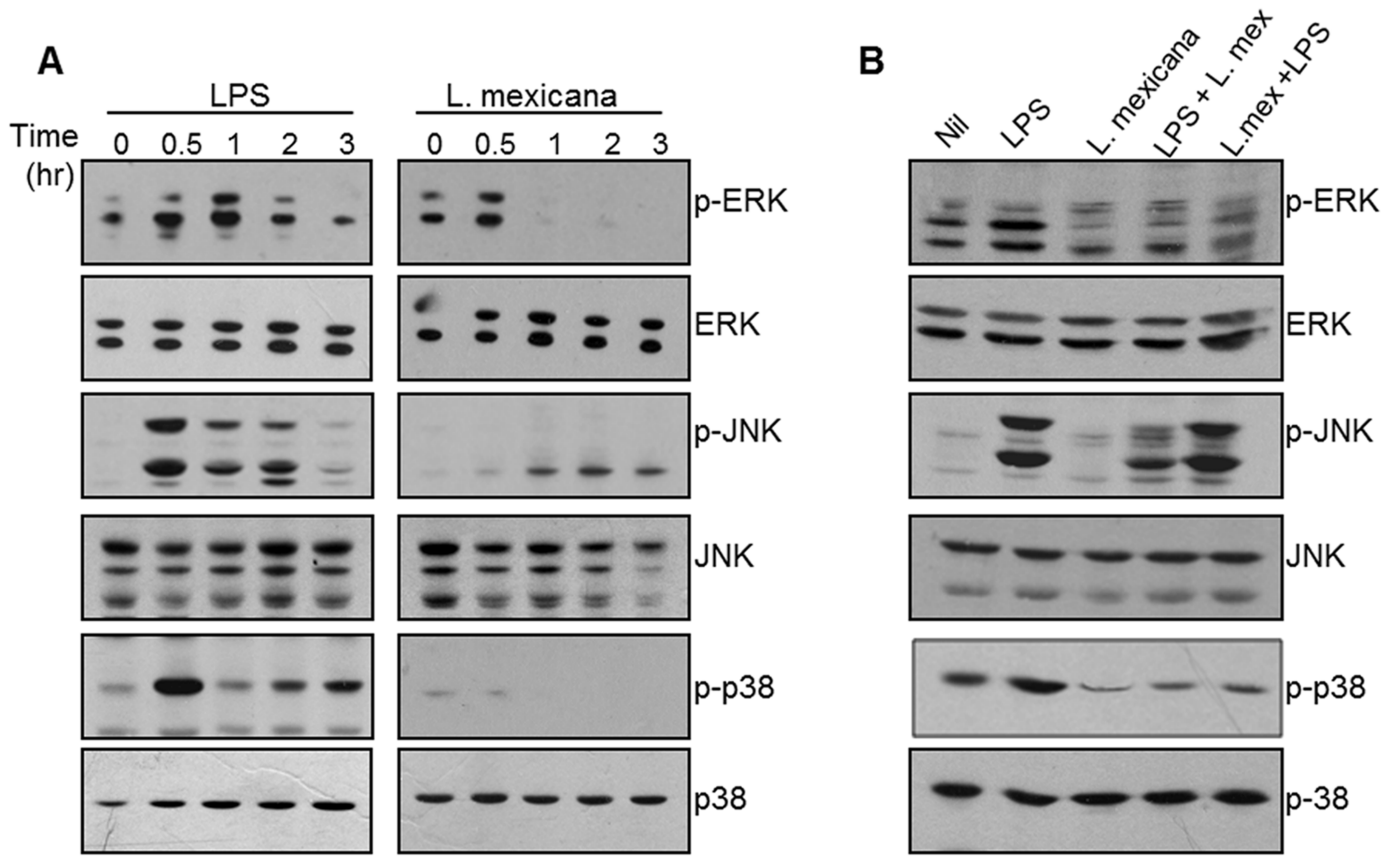
*L. mexicana* promastigotes inhibit MAPK phosphorylation. (**A**) Western blot analysis of phosphorylation of ERK, JNK and p38 proteins in cell lysates. DC2.4 cells were stimulated with 100 ng/ml of LPS or infected with *L. mexicana* promastigotes for 0.5, 1, 2, and 3 hr. (**B**) WB analysis of phosphorylation of ERK, JNK and p38 proteins in total cell lysates. DC 2.4 cells were stimulated for 0.5 hr with LPS (100 ng/ml), infected with *L. mexicana* promastigotes for 1 hr, stimulated with LPS for 0.5 hr and then infected for 1 hr, or infected for 1 hr and then stimulated with LPS for an additional 0.5 hr. In all the cases DCs were infected in a 1∶20 cell parasite ratio. One representative experiment of three is shown.

Protein phosphorylation though MAPKs plays an essential role in many cellular functions [24]. Therefore, we investigated whether *Leishmania* infection could affect the phosphorylation of MAP kinases (ERK, p38 and JNK) in the DC2.4 cell line. Activation of MAP kinases in DCs by LPS is well documented and varies depending on the DC's origin [25], [26]. However, we wanted to determine whether LPS stimulation could induce MAPK phosphorylation in the DC2.4 cells. Western blot analysis (Figure 2A) revealed that phosphorylation of ERK, p38 and JNK was increased as soon as 30 minutes post-stimulation. On the other hand, infection with *L. mexicana* promastigotes inhibited basal phosphorylation of ERK, JNK and p38, without affecting total protein expression (Figure 2A right panels).

To analyze the impact of *Leishmania* infection on MAP kinase phosphorylation induced by LPS, we designed experiments where the LPS stimulation was performed either before or after infection. Based on the LPS stimulation and *L. mexicana* infection time-courses, we chose 30 minutes of stimulation with LPS and 60 minutes of *L. mexicana* infection as the optimal time points for analysis. Figure 2B shows that infection with *L. mexicana* after LPS stimulation reduced phosphorylation of ERK, JNK and p38. The same reduction on ERK and p38 phosphorylation was observed when DCs were infected before LPS stimulation; however, JNK phosphorylation was not affected in this condition.

### Inhibition of translocation of transcription factors (TFs)

**Figure 3:**
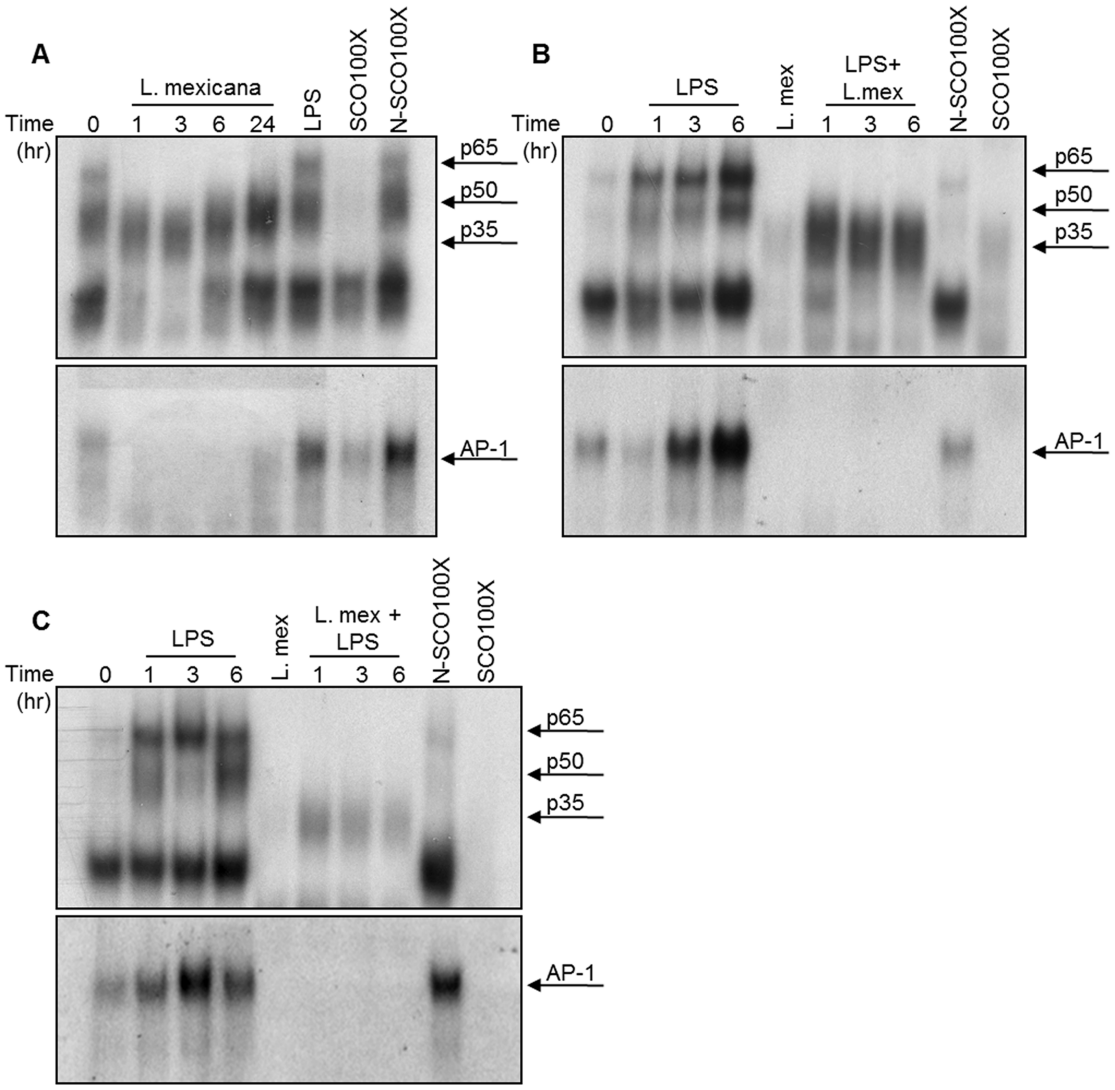
*L. mexicana* promastigotes abrogate nuclear translocation of transcription factors. (**A**) DC 2.4 cells were infected with *L. mexicana* promastigotes for 1, 3, 6 and 24 hr and nuclear proteins were subjected to EMSA for NF-κB (p65/p50) or AP-1 (**B**) DC2.4 cells were stimulated with 100 ng/ml of LPS for 1, 3 and 6 hr or stimulated for 3 hr with LPS and infected with *L. mexicana* promastigotes for 1, 3 and 6 hr. Nuclear proteins were subjected to EMSA for NF-κB (p65/p50) or AP-1. (**C**) DC 2.4 cells were infected with *L. mexicana* for 1, 3 and 6 hr and then stimulated with 100 ng/ml of LPS for an additional 3 hr, nuclear proteins were subjected to EMSA for NF-κB (p65/p50) or AP-1. Consensus oligonucleotides nonspecific competitor (NSCO) and specific competitor (SCO) were used in a 100× molar excess. One representative experiment of three is shown.

We have previously demonstrated that different *Leishmania* species are able to abrogate the activity of important TFs in macrophages such as STAT 1 [16], NF- κB [27], and AP-1 [15]. Here, we investigated the effect of *L. mexicana* promastigotes on these TFs, either alone or in combination with LPS. Infection of DC 2.4 cells led to a reduction in the nuclear translocation of both AP-1 and NF-κB as soon as 1 hr post-infection (Figure 3A). In contrast, stimulation of DCs with LPS conferred them the ability to translocate AP-1 and NF-κB (Figure 3B and 3C). Parallel experiments of LPS-stimulated DCs before infection (Figure 3B) or infected and then stimulated with LPS (Figure 3C) showed that, in either case, infection prevented the translocation of TFs. However, in the case of NF-κB, we observed the appearance of a lower band, known as p35, in cells that were stimulated with LPS before infection. This p35 subunit is able to dimerize with other NF-κB subunits and then translocate into the nucleus to bind DNA [27].

### *L. mexicana* promastigotes affect DC maturation by inhibiting the expression of MHC and co-stimulatory molecules

**Figure 4:**
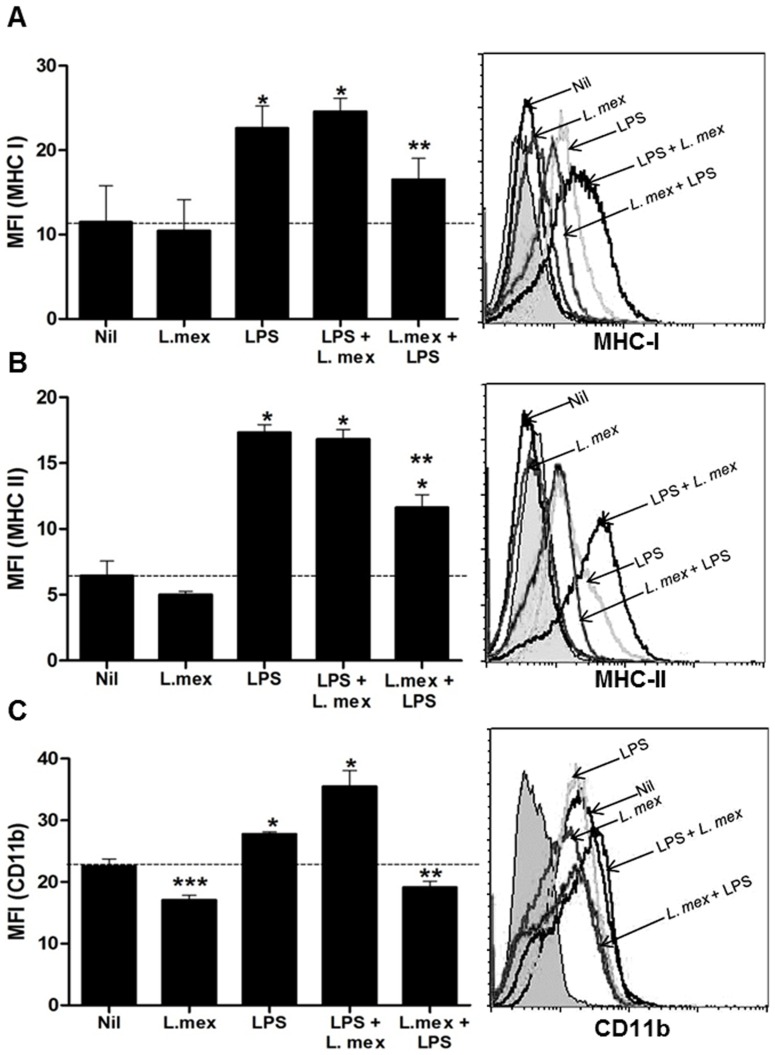
Decreased expression of antigen presentation molecules in *L. mexicana*-infected DCs. DC2.4 cells were stimulated only with LPS (100 ng/ml) for 24 hr or LPS-stimulated before or after infection with *L. mexicana* promastigotes for 24 hr. Collected cells were stained with (A) anti-MHC I (Cy5), (B) anti-MCH II (Cy5) and (C) anti-CD11b (FITC). After staining, cells were analyzed by flow cytometry. (*) denotes P<0.05 between groups compared with uninfected DCs; (**) denotes P<0.05 between groups compared with LPS-matured DCs; (***) denotes P<0.05 between uninfected DCs and *L. mexicana*-infected DCs. The dashed line denotes the basal expression of the molecules in uninfected cells. Data pooled from three different experiments is shown.

**Figure 5:**
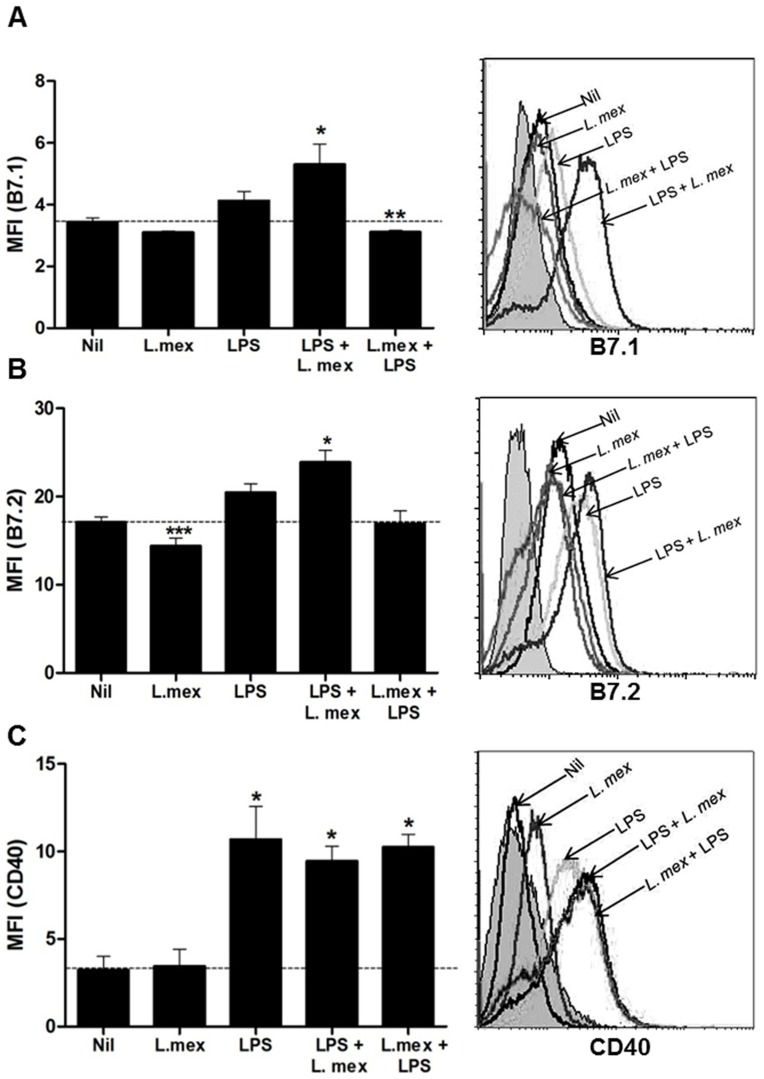
Decreased expression of co-stimulatory molecules in *L. mexicana*-infected DCs. DC2.4 cells were stimulated only with LPS (100 ng/ml) for 24 hr or LPS-stimulated before or after infection with *L. mexicana* promastigotes for 24 hr. Collected cells were stained with (**A**) anti-B7.1 (PE), (**B**) anti-B7.2 (FITC) and (**C**) anti-CD40 (PE). After staining, cells were analyzed by flow cytometry. (*) denotes P<0.05 between groups compared with uninfected DCs; (**) denotes P<0.05 between groups compared with LPS-matured DCs; (***) denotes P<0.05 between uninfected DCs and *L. mexicana*-infected DCs. The dashed line denotes the basal expression of the molecules in uninfected cells. Data pooled from three different experiments is shown.

DCs are professional APCs, which, in an immature state, are able to uptake and process a wide variety of antigens [9]. The maturation process requires the increased expression of MHC molecules, as well as co-stimulatory molecules. Therefore, we were interested in investigating whether abrogation of the signaling pathways by *L. mexicana* could alter DC maturation and APC functions. In this regard, we infected DC2.4 cells with *L. mexicana* or stimulated them with LPS. Thereafter, we monitored the expression of MHC-I, MHC-II, B7.1, B7.2, CD40 and CD11b by Flow Cytometry. *L. mexicana* infection significantly decreased the basal levels of CD11b and B7.2, and slightly decreased MHC II and B7.1 expression (Figures 4 and 5). To determine the maturation status of the cells, we stimulated DCs for 24 hr with LPS. Increased expression levels of MHC I, MHC II, CD11b, B7.1, B7.2, and CD40 were detected in response to LPS. We also evaluated the expression of these molecules after 48 hr of LPS stimulation and we did not observe a significant change on the maturation markers, compared with the levels expressed at 24 hr (Data not shown). To determine the effect of *Leishmania* infection on DC maturation, DCs were matured with LPS for 24 hr and then infected. Under these conditions, *L. mexicana* infected-DCs showed similar levels of maturation markers compared to cells stimulated only with LPS. In contrast, infection for 24 hr prior to LPS stimulation significantly reduced the levels of MHC I, MHC II, CD11b and B7.1 in comparison with LPS-matured cells (Figures 4 and 5), suggesting that addition of LPS after infection is not sufficient to reverse the suppressing effect of *L. mexicana* promastigotes on the expression of these molecules. Interestingly, CD40 expression was increased in LPS-matured DCs, and its expression was not affected by *L. mexicana*. The summarized results of the expression of these molecules in the DC2.4 cells can be observed in Table 1 (% of MFI increase over non-infected control).

**Table 1:** Change in medium fluorescence intensity (MFI; %) upon *Leishmania* infection.

	MHC-I	MHC-II	CD11b	B7.1	B7.2	CD40
**Nil**	100	100	100	100	100	100
***L. mex***	91.9±31.5	78.1±37.5‡	75.8±3.2* ‡	89.8±1.2* ‡	84.2±5.3* ‡	106.3±30‡
**LPS**	198.3±22.8*	270.3±9.4*	123.1±1.7*	119.8±8.4	119.9±5.9*	334.3±56*
**LPS+ ** ***L. mex***	215.8±13.6*	262.5±9.4*	157.4±11.3* ‡	153.9±19*	140.4±7.6*	295.6±26*
***L. mex*** ** +LPS**	145±21.9‡	181.3±15.6* ‡	84.6±4.4* ‡	89.9±3.5* ‡	107.6±9.4	320.6±22*

Data obtained from a pool of three different experiments (made in duplicate). Change in the % of MFI was compared with Nil (100%). An unpaired T test with Welch correction analysis was used.

*P≤0.05 compared to Nil,

‡ P≤0.05 compared to LPS-stimulated cells.

+/− represents the standard error (SEM).

**Figure 6:**
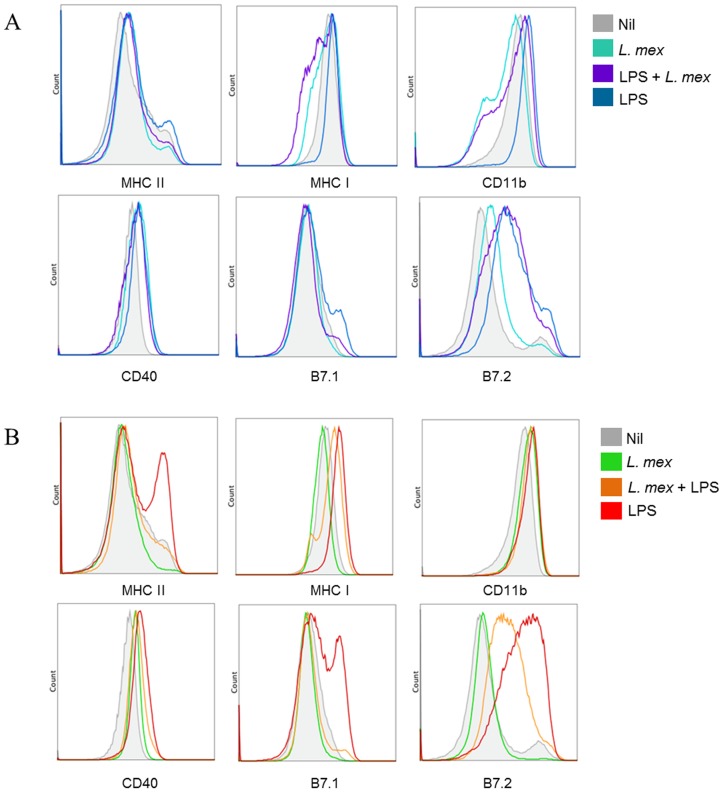
*L. mexicana* promastigote-infected BMDCs have decreased expression of antigen presentation and co-stimulatory molecules. BMDCs were stimulated with LPS (100 ng/ml) for 24 hr or LPS-stimulated before (**A**) or after (**B**) an 18 hr infection with *L. mexicana* promastigotes. Collected cells were stained with the indicated antibodies. After staining, cells were analyzed by flow cytometry. One representative experiment of three is shown.

To examine the ability of *L. mexicana* to suppress DC activation in primary DCs, we performed similar experiments using bone marrow-derived DCs (BMDCs). We found that *L. mexicana* infection of BMDCs also inhibited LPS-induced activation of MHC and co-stimulatory molecules. The results showed similar patterns of expression to those observed using DC2.4 cells. As shown in Figure 6, Infection with *L. mexicana* promastigotes reduced the expression of MHC-II, B7.1 and B7.2 (Figures 6A and 6B). Additionally, LPS stimulation prior to infection did not increase expression of these molecules (Figure 6A). Importantly, infection prior to LPS stimulation also inhibited expression of the same molecules (Figure 6B). Similarly to what we observed in DC2.4 cells, expression of CD40 was not altered in BMDC cells in either condition of infection.

### *Leishmania* parasites reduce the capacity of DCs to stimulate T cell responses

**Figure 7:**
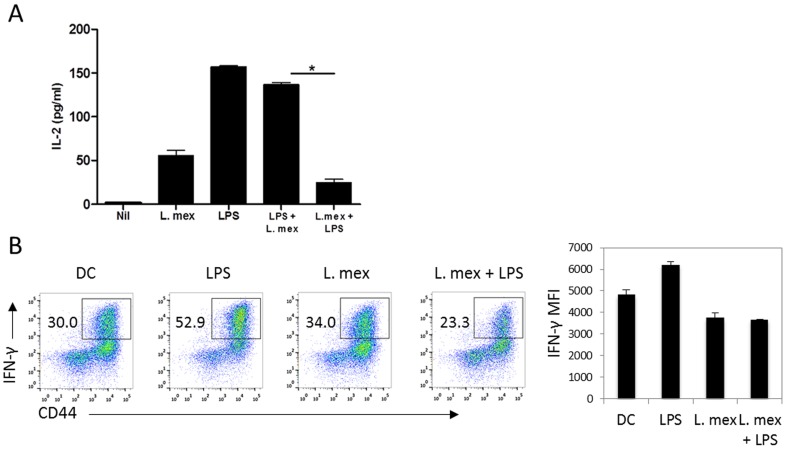
*L. mexicana* promastigotes inhibit the ability of DCs to stimulate Th-1 cell responses. (**A**) DC2.4 cells were stimulated with LPS, infected with *L. mexicana* promastigotes for 24 hr or stimulated, before or after 24 hr of infection, with 100 ng/ml of LPS. Afterwards, cells were washed and loaded with 2 mg/mL of chicken OVA, for 2 hr, and then incubated ON with 50,000 OVA-specific T cells. IL-2 was measured in supernatants by ELISA. One representative experiment of five is shown. (**B**) Expression of IFN-γ by OT-II T cells co-cultured for 3 days with primary BMDCs that were either uninfected or infected with *L. mexicana* for 6 hours, with or without LPS stimulation (100 ng/mL) ON. After three days of culture, T cells were stimulated with PMA and ionomycin and intracellular IFN-γ was determined by ICS/flow cytometry. MFI of IFNγ+ population is shown. One representative experiment of two is shown.

The ability of DCs to activate naïve T cells depends on their maturation stage [9], [28]. In this study, we examined the effect of *L. mexicana* infection on the ability of DCs to stimulate T cell responses. First, we studied IL-2 production by ovalbumin-specific T cells in response to DC2.4 cells pulsed with OVA and stimulated/infected with LPS, *L. mexicana* or in combination. IL-2 production by T cells was significantly reduced when cultured with *Leishmania*-infected DCs, compared to LPS-stimulated DCs (Figure 7A). In contrast, *Leishmania* infection did not have any effect on LPS-induced T cell activation, as IL-2 production by T cells co-cultured with LPS-matured DCs and then infected for 24 hrs was similar to that of cultures of T cells with DCs stimulated only with LPS. However, when, T cells were co-cultured with *L. mexicana*-infected DCs prior to stimulation with LPS, they produced ∼85% less IL-2, suggesting that *L. mexicana* impairs the ability of DCs to stimulate T cell responses (Figure 7A).

To examine this further, we analyzed the ability of primary BMDCs to stimulate primary antigen-specific T cell responses. LPS stimulated DCs will promote the differentiation of naïve T cells to the Th1 lineage. Furthermore, Th1 responses are necessary for the control of *Leishmania* infection. Uninfected DCs and those infected with *L. mexicana* were treated with or without LPS, pulsed with ovalbumin and examined for their ability to promote the differentiation of naïve OT-II cells to Th1 cells. LPS stimulation of DCs alone resulted in the production of Th1 cells, as measured by the production of IFNγ (Figure 7B). However, when cells were infected with *Leishmania*, they did not induce Th1 cell development beyond the levels of control DCs. When LPS-activated DCs were pre-infected with *L. mexicana*, they also failed to induce Th1 cell development beyond the levels of control DCs, demonstrating that *L. mexicana* infection inhibits Th1 development by LPS-activated DCs.

### *L. mexicana* promastigotes reduce IL-12 mRNA expression in DCs

**Figure 8:**
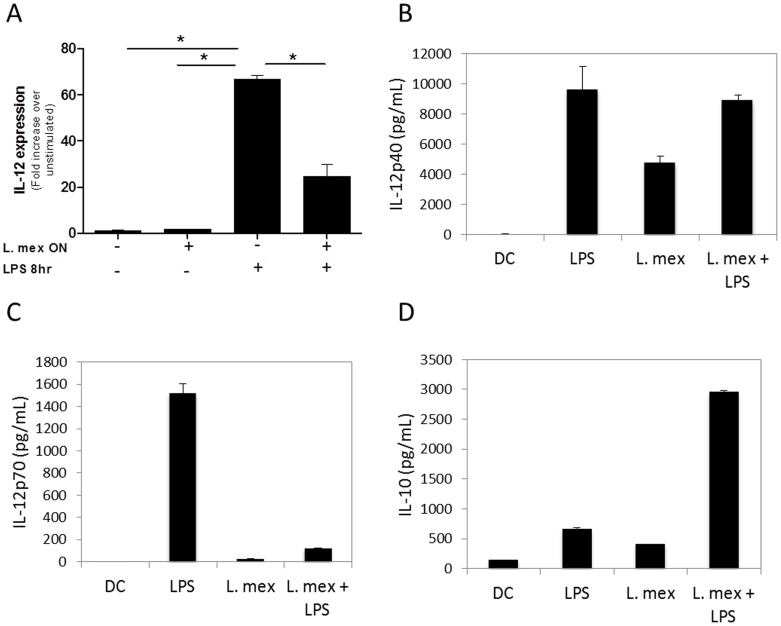
*L. mexicana* promastigotes alter the cytokine production profile of DCs. (**A**) DC2.4 cells were infected with *L. mexicana* and then stimulated for 8 hrs with LPS. mRNA was extracted and IL-12 mRNA expression was analyzed by RT-PCR. (*) denotes P<0.05 between specific groups. Data pooled from three experiments is shown. (**B–D**) Primary BMDCs were infected with *L. mexicana* or left in media for 6 hrs, with or without LPS (100 ng/mL) ON. Levels of secreted IL-12p40, IL-12p70, and IL-10 were measured by ELISA. One representative experiment of three is shown.

IL-12 produced by DCs plays a critical role in the development of CD4^+^ Th1 cells, which confer resistance against *L. major*-infection in mice [29]. Furthermore, the transcription factors AP-1 and NF-κB are responsible for IL-12 production [30], [31]. Therefore, we investigated whether infected DC 2.4 cells had an altered capacity to produce IL-12. IL-12 p40 expression peaked 8 hrs post-stimulation with LPS (Figure 8A). *L. mexicana* infection did not induce significant levels of IL-12 expression compared to uninfected cells (Figure 8A). DC infection after LPS stimulation did not impair IL-12 mRNA levels; however, when DCs were first infected and then stimulated with LPS, IL-12 mRNA expression was reduced more than 30 fold, indicating that *L. mexicana* infection renders DCs refractory to further LPS stimulation, in line with the demonstration that LPS-signaling in infected cells was altered.

We examined the regulation of IL-12 production by *L. mexicana* in primary BMDCs. DCs were stimulated with or without LPS, prior to infection with *L. mexicana*. In primary BMDCs, we found that *L. mexicana* infected DCs produced IL-12p40 and low levels of IL-12p70 (Figures 8B and 8C). However, *L. mexicana* infection had minimal effects on the ability of LPS-activated DCs to produce IL-12p40, but drastically suppressed their ability to produce IL12p70. Together, these results demonstrate that *L. mexicana* infection impairs IL-12 production in DCs, which inhibits their ability to promote Th1 cell development.

### *L. mexicana* promotes IL-10 production by DCs

Our results have demonstrated that *L. mexicana* can interfere with DC activation, even in response to a strong stimulus, such as LPS. We hypothesized that the decrease in DC activation could also be due to increased production of the suppressive cytokine IL-10, as IL-10 is known to suppress the expression of co-stimulatory molecules and cytokine production. IL-10 production was examined in BMDC cells stimulated with or without LPS, prior to infection with *L. mexicana*. We found that *Leishmania* infection resulted in a modest increase in IL-10 production, similar to that induced by LPS stimulation (Figure 8D). However, DCs infected with *L. mexicana* and then stimulated with LPS, produced much higher levels of IL-10.

Together, our data suggest that alterations in DC maturation and cytokine production upon infection with *L. mexicana* promastigotes involve activation of PTPs that can dephosphorylate important MAP kinases and inhibit nuclear translocation of AP-1 and NF-κB, resulting in DCs with a suppressed activation phenotype, characterized by decreased expression of MHC and costimulatory molecules, decreased IL-12 production and increased production of IL-10

## Discussion

In addition to macrophages, other cell types can become hosts for *Leishmania*, including DCs which have a critical role in priming the adaptive immune response [4]–[7]. In order to be able to present antigens and prime the adaptive immune response, DCs have to undergo a maturation process. The antigen sampling, migratory capacities and cytokine production by DCs, effectively allow naïve T cells to become activated by antigens and differentiate into Th1 cells [32]. Taking into account that DCs are the link between the innate and adaptive immune responses and their capacity to uptake *Leishmania* parasites, we sought to determine the effect of infection with *L. mexicana* promastigotes on DC2.4 cells and BMDC functions and signaling pathways.

In the present work, we have attempted to discern the impact of *L. mexicana* infection on antigen presentation by DCs in two different situations: first, infection of previously matured DCs and second, infection of DCs followed by LPS stimulation, to drive further maturation. Our data showed that both infected DCs from the cell line DC2.4 or primary DCs (BMDCs) that received the signal to induce their maturation (LPS) after infection by *L. mexicana* promatigotes, were not able to mature and showed lower expression of MHC class I and class II and co-stimulatory molecules, such as B7.1 and B7.2. On the other hand, LPS clearly induced DC maturation by increasing the expression of the aforementioned molecules and promoting T cell activation. Furthermore, infection of LPS-matured DCs did not affect their expression of co-stimulatory molecules. Besides analyzing the expression of MHC and co-stimulatory molecules, we assessed the impact of infection on the capacity of DCs to present antigen to OVA-specific T cells. LPS-matured infected DCs were able to activate T cells. These data suggest that LPS stimulation prior to infection might prime DCs to produce more IL-12 after a second stimulus (*L. mexicana*). However, T-cells co-cultured with DCs infected with *Leishmania* prior to LPS stimulation exhibited a reduced capacity to produce IL-2, supporting the notion that *L. mexicana* interferes with the maturation and antigen presentation process. *Leishmania* infection could be affecting many of the DC's functions, so we examined the ability of primary DCs to promote the development of Th1 responses. Consistent with results from DC2.4 cells, *Leishmania* infected BMDCs stimulated with LPS showed reduced IL-12p70, increased IL-10 and a diminished ability to promote Th1 responses. Collectively, our results demonstrate that *Leishmania* interferes with proper DC activation and function.

Regarding antigen presentation and accessory functions and in agreement with our findings, Liu *et al.* showed that *L. major* LPG is involved in the inhibition of DC maturation and IL-12 production [33]; in addition to this study, Boggiatto *et al.* showed that infection of BMDCs (from C3HeB mice) with amastigotes of *L. amazonensis* reduced the expression of CD40 and production of IL-12 [34]; furthermore, Neves *et al.* demonstrated that *L. infantum* promastigotes decreased the expression of CD40 and B7.2 (CD86), as a result of the impairment of the NF-κB signaling pathway [35]. In contrast, De Trez *et al.* showed that *L. donovani* increases the maturation and migration capacity of DCs from both BALB/c and C57BL/6 mice [36]. These data suggest that DC responses differ according with the *Leishmania* species and the parasite stage used for infection. Moreover, all these studies have shown that the evasion mechanisms used by the parasite are very diverse, since it has been observed that the parasite can activate or deactivate signaling cascades to its convenience, in order to survive inside their host.

In order to better understand the mechanisms underlying the down regulation of the antigen presentation capacity of DCs, we investigated the phosphorylation of MAP kinases and nuclear translocation of transcription factors such as AP-1 and NF-κB. Signal transduction through MAPK plays an important role in many cellular responses and it is well known that ERK, JNK and p38 are *Leishmania* targets [37]. Our results showed that infection of DCs with *L. mexicana* promastigotes prior to stimulation with LPS led to inhibition in the phosphorylation of p38 and ERK kinases, but not JNK. In accordance with the reduced phosphorylation of MAPKs, by in-gel and pNPP assays, we observed that as soon as 30 min post-infection, a rapid activation of host's phosphatases was induced. Although this amount of time is not enough for the parasite to get internalized, we have previously reported that the parasite's glycoprotein gp63 is able to enter the cells independently of the parasite, through lipid rafts, and this happens as soon as 5 minutes post-infection. Once inside the cell, gp63 rapidly activates the host's PTPs [18].

It is known that, in DCs of the D1 cell line, LPS activates the ERK pathway, playing a role in DC maturation and in preventing apoptosis after maturation [26]. In addition, p38 and the PI3 kinase pathways are involved in LPS-induced maturation of human monocyte-derived dendritic cells [25]. Our findings suggest that, in an immature state, infected DCs are unable to phosphorylate JNK, p38 and ERK. However, *Leishmania* does not suppress JNK phosphorylation in LPS-matured DCs. The unimpaired LPS-triggered JNK phosphorylation could somehow benefit the activation of molecules that help the parasite to survive within the host cell. Furthermore, JNK phosphorylation could imply that the expression of other co-stimulatory molecules might not be affected by *Leishmania* infection, as Nakahara *et al.* reported that the JNK pathway is involved in the maturation of DCs by inducing expression of CD54 and CD83 [38], [39]. However, in DCs infected prior to LPS stimulation, a normal expression of CD54 and CD83 may not be sufficient to activate T cells, as we observed a significant decrease in IL-2 production by T cells under these conditions.

We also analyzed the expression of CD40. CD40 is an important co-stimulatory molecule involved in antigen presentation [40]. Even though CD40 expression was not altered by *Leishmania* infection, it could not compensate for the diminished expression of other co-stimulatory molecules (B7.1, B7.2), as we observed a significant decrease on IL-2 production by T cells. Interestingly, the normal expression of CD40 in infected, LPS- matured cells, can be explained by the fact that p38 and ERK are not involved in the expression of CD40, but they are necessary for the expression of MHC I, MHC II, B7.1 and B7.2 molecules in DCs [25], [41].

This study further showed that nuclear translocation of transcription factors was also inhibited in infected DCs both pre-treated and post-treated with LPS. In the particular case of AP-1, nuclear translocation of this important transcription factor was completely suppressed by *Leishmania* and JNK phosphorylation was not sufficient to induce its activation. One possible explanation of the latter event could be that activation of ERK, which is also up-stream of AP-1activation, was inhibited by the parasite. We do not rule out the possibility that *Leishmania* infection could inactivate other signaling pathways involved in the activation of AP-1, as we have shown that *Leishmania* affects other subunits that form AP-1 [15]. In the case of NF-κB, we found that nuclear translocation of this TF is abrogated. However, we observed the appearance of the cleavage fragment p35 in DCs stimulated with LPS prior to infection. The p35 subunit is able to dimerize with other NF-κB subunits and bind specific DNA sequences [27]. In parallel with the inhibition of the translocation of the TF we observed that IL-12 production was also affected by *L. mexicana* promastigotes infection. The lower production of IL-12 could be contributing to the diminished T cell activation, as it is known that IL-12 is crucial for T-cell and B-cell activation during the formation of the immunological synapse [42] and to activate other co-accessory cells such as NK cells [43].

Despite the fact that in this study we have clearly demonstrated that *L. mexicana* promastigotes alter the signaling pathways and antigen presentation capacity of DC2.4 cells, we do not preclude the possibility that some other functions could also be affected during infection, such as phagosome maturation and modulation in the chemokines and cytokines expression (several studies have shown contradictory results); it is noteworthy to mention, that some of these functions are under study in our laboratory, to further strengthen our findings.

Collectively, this present study has permitted to establish that, as in macrophages, *L. mexicana* promastigotes are able to rapidly abrogate important signaling pathways that affect the maturation of dendritic cells (from cell line and primary cells), therefore affecting its capacity to induce a proper adaptive immune response during the initial stage of infection and this in order to better survive and propagate within its host.

## Supporting Information

Figure S1 ***L. mexicana*** **promastigote-infected BMDCs.** BMDCs (0.1×10^6^/ml) were plated on coverslips and infected for 18 hr with *L. mexicana* promastigotes at the indicated ratio (parasite-cell).
(TIF)

Figure S2 **In-gel assay of DC2.4 cells and B10R macrophages infected with** ***L. mexicana*** **.** (**A**) In-gel PTP assay of *Leishmania*-infected DC: DCs from cell line (DC2.4), uninfected, LPS stimulated (100 ng/ml/1 hr), infected with *L. mexicana* promastigotes (20∶1) for up to 6 hrs, and incubated with parasite lysates corresponding to 10^7^ and 10^8^ *Leishmania* promastigotes. (**B**) In-gel PTP assay of *Leishmania*-infected B10R macrophages and Western blot showing levels of gp63 detected in infected cells, versus the amount corresponding to 10^7^ promastigotes.
(TIF)
