# Supplementary figures and images for: Impact of Leishmania mexicana Infection on Dendritic Cell Signaling and Functions
Source: PLoS Negl Trop Dis. 2014 Sep 25;8(9):e3202. doi: 10.1371/journal.pntd.0003202 (PMC4177750; doi:10.1371/journal.pntd.0003202)

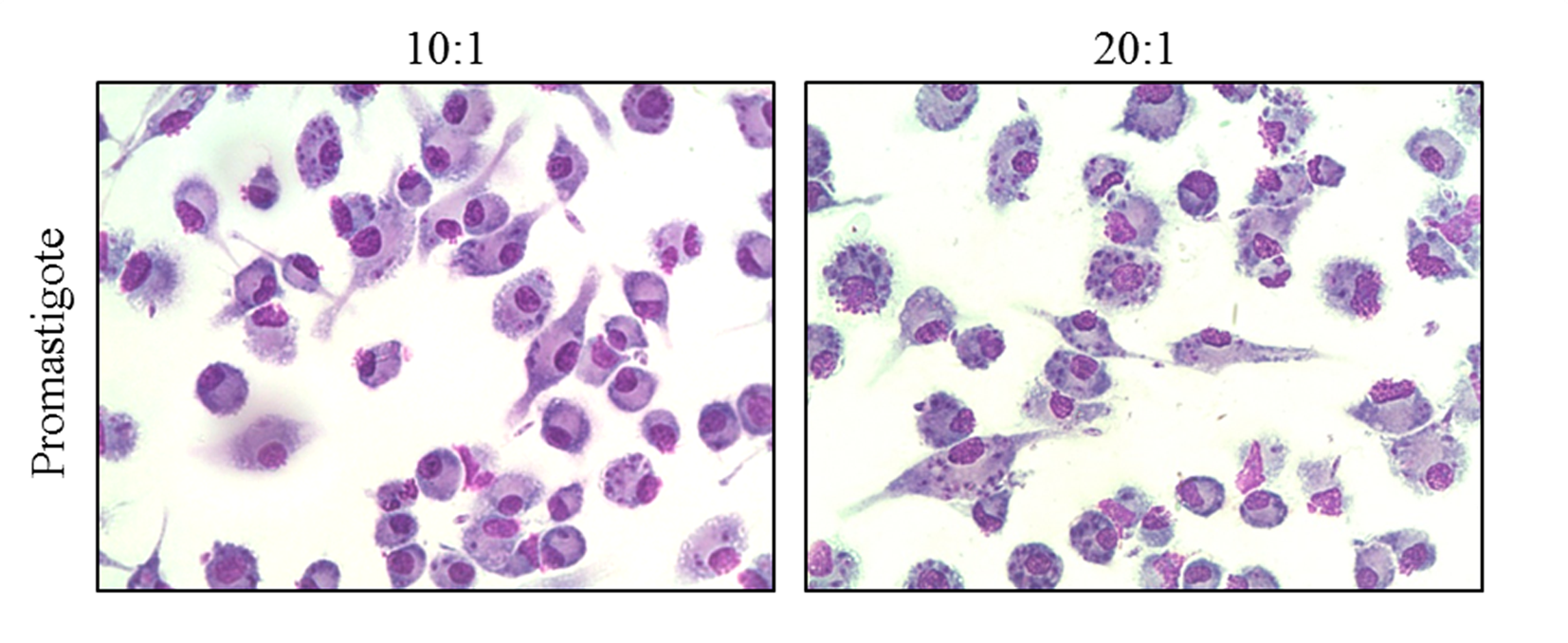

Supplement: Figure S1 — L. mexicana promastigote-infected BMDCs. BMDCs (0.1×10^6^/ml) were plated on coverslips and infected for 18 hr with L. mexicana promastigotes at the indicated ratio (parasite-cell). (TIF) [file pntd.0003202.s001.tif]

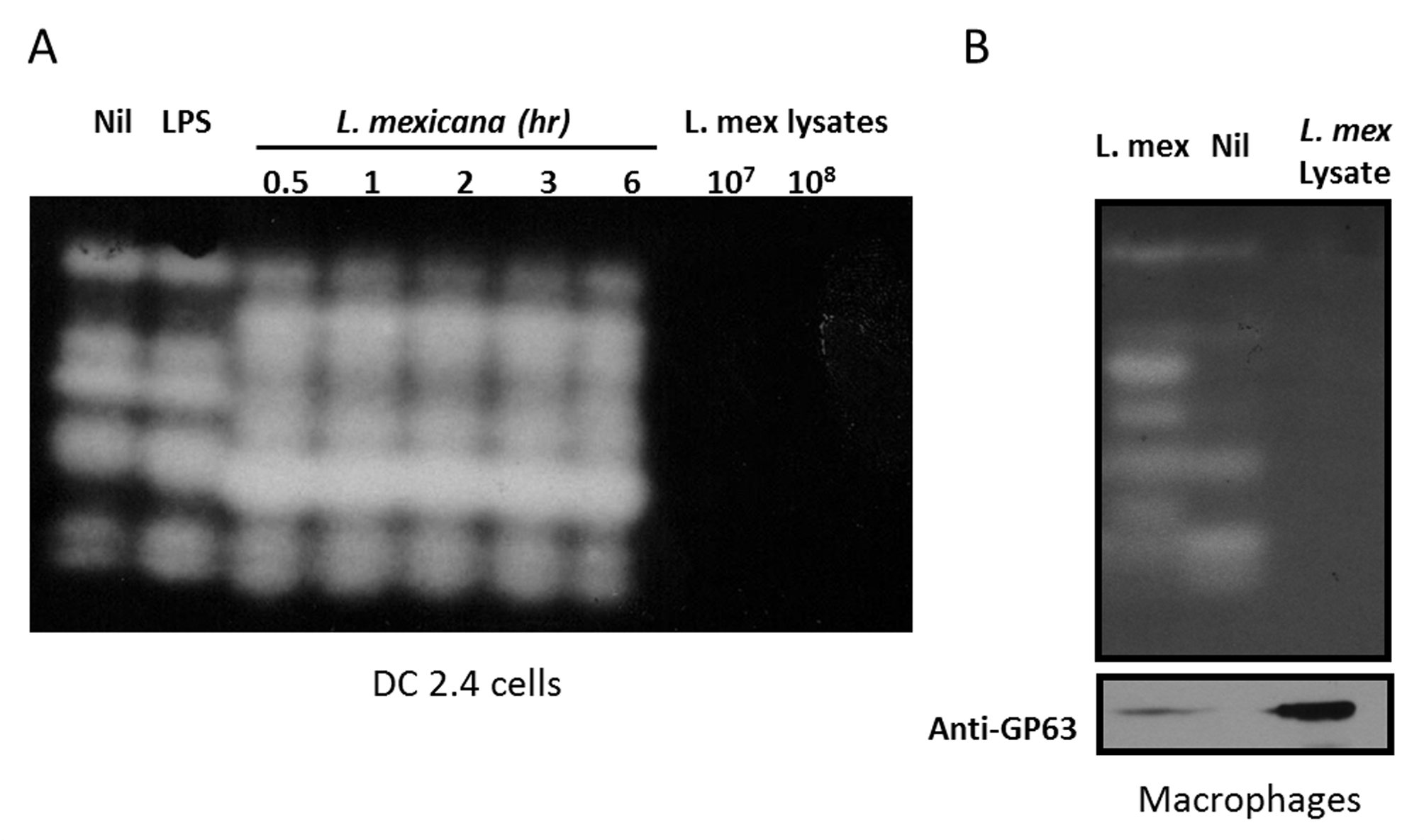

Supplement: Figure S2 — In-gel assay of DC2.4 cells and B10R macrophages infected with L. mexicana . (A) In-gel PTP assay of Leishmania-infected DC: DCs from cell line (DC2.4), uninfected, LPS stimulated (100 ng/ml/1 hr), infected with L. mexicana promastigotes (20∶1) for up to 6 hrs, and incubated with parasite lysates corresponding to 107 and 108 Leishmania promastigotes. (B) In-gel PTP assay of Leishmania-infected B10R macrophages and Western blot showing levels of gp63 detected in infected cells, versus the amount corresponding to 107 promastigotes. (TIF) [file pntd.0003202.s002.tif]
